# Supplementary material for: Voltage-driven gigahertz frequency tuning of spin Hall nano-oscillators
Source: Nat Commun. 2022 Jun 30;13:3783. doi: 10.1038/s41467-022-31493-z (PMC9246901; doi:10.1038/s41467-022-31493-z)
Supplement: Supplementary file 1 — Supplementary Information [file 41467_2022_31493_MOESM1_ESM.pdf]

Supplementary Information

Voltage-driven gigahertz frequency tuning of spin Hall nano-oscillators

Jong-Guk Choi[1†], Jaehyeon Park[2†], Min-Gu Kang[1], Doyoon Kim[3], Jae-Sung Rieh[3], Kyung-Jin Lee[2], Kab-Jin Kim[2★] and Byong-Guk Park[1★]

[1] *Department of Materials Science and Engineering, KAIST, Daejeon 34141, Korea*

[2] *Department of Physics, KAIST, Daejeon 34141, Korea*

[3] *School of Electrical Engineering, Korea University, Seoul 02841, Korea*

[†] *These two authors equally contributed to this work.*

★ **Correspondence to:** kabjin@kaist.ac.kr (K.-J.K.) and bgpark@kaist.ac.kr (B.-G.P.)

- Contents -

Note 1. Retention of voltage-driven state

Note 2. Fast operation of voltage controlled magnetic anisotropy at room temperature

Note 3. ST-FMR spectra depending on the polar angle of magnetic field

Note 4. ST-FMR spectra for various frequencies

Note 5. Origin of voltage controlled magnetic anisotropy and damping

Note 6. Voltage-driven frequency modulation in Co/Ni sample with a different thickness

Note 7. Gate voltage effect on the current-induced SOT

Note 8. Nonlinearity coefficient depending on perpendicular magnetic anisotropy

Note 9. Comparison of auto-oscillation frequency and FMR frequency

Note 10. Threshold current for current-induced magnetization auto-oscillation

Note 11. Gate-induced leak current

Note 1. Retention of voltage-driven state

To demonstrate the retention of the voltage-driven state, we measured how long the voltage-controlled frequency lasts after turning off the gate voltage. Supplementary Figure 1 shows auto-oscillation frequency as a function of time for our Co/Ni sample. Here, we used the same experimental conditions as Figs. 3 and 4 of the main text and the driving current of 3.1 mA was used. The result shows that the frequency is almost constant up to 10^4 seconds both for +5 V (red) and for -5 V (black). We note that this retention time is comparable to the characteristic time scale in previously reported memristive devices [S1-S3]. This indicates that the voltage-driven frequency modulation can be utilized in long term plasticity of the neuromorphic device [S4,S5].

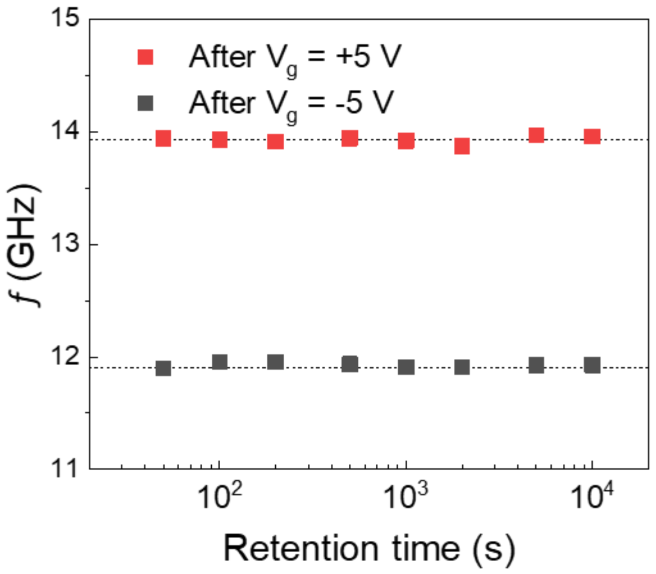

Supplementary Figure 1| Retention of voltage-driven state. Temporal variation of auto-oscillation frequency after turning off the gate voltage of +5 V (red) and -5 V (black) for Ta (3 nm)/Pt (5 nm)/[Co (0.4 nm)/Ni (0.6 nm)]$_7$/Co (0.4 nm)/AlO_x (2 nm)/Ta(3 nm)/ZrO2(40 nm) sample

Note 2. Fast operation of voltage controlled magnetic anisotropy at room temperature

In the main manuscript, we used a 40 nm thick gate oxide (ZrO_2) for which a gate voltage needs to the applied for a few seconds to minutes at 150°C to observe a sizable electric field effect. We below show that this is not a fundamental limit of our device but could be further improved by materials engineering. To verify this argument, we tested another sample of Ta(3 nm)/Pt(5 nm)/[Co(0.45 nm)/Ni(0.6 nm)]$_3$/Co(0.45 nm)/AlO_x(2 nm) structure with a thin gate oxide of ZrO_2 (5 nm). The sample was patterned into a Hall bar device with a 10 μm × 10 μm Hall cross, which is the same structure as the VCMA test sample shown in Fig. 1c of the main text. Supplementary Figure 2 shows the anomalous Hall resistance (R_{xy}) as a function of perpendicular magnetic field (B_z), demonstrating that the coercivity of the sample is reduced by a gate voltage (V_g = 20 V) applied at room temperature for 1 ms. Furthermore, we showed in the previous report [S6] that the operation speed of the VCMA effect at room temperature decreased to 20 μs by introducing double gate oxide of TiO_2(2 nm)/ZrO_2(5 nm) structure. This is because a larger electric field is applied for a thinner gate oxide and the TiO_2 has high oxygen ion mobility. These results indicate that the operation time in our sample can be further improved by material engineering.

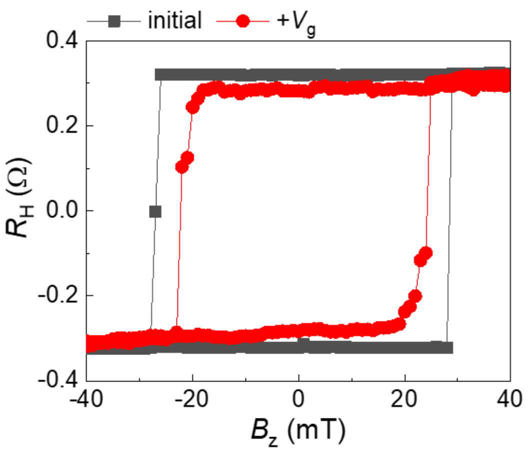

Supplementary Figure 2| Voltage-controlled magnetic anisotropy with a thin gate oxide. Anomalous Hall resistance (R_{xy}) as a function of perpendicular magnetic field (B_z) in a Ta (3 nm)/Pt (5 nm)/[Co (0.45 nm)/Ni (0.6 nm)]$_3$/Co (0.45 nm)/AlO_x (2 nm)/ZrO_2(5 nm) sample when applying a V_g of +20 V for 1 ms at room temperature.

Note 3. ST-FMR spectra depending on the polar angle of magnetic field

To confirm that the two-magnon scattering (TMS) occurs in our sample, we conducted the ST-FMR measurement with different polar angles of magnetic fields (θ) in the Ta (3 nm)/Pt (5 nm)/[Co (0.45 nm)/Ni(0.6 nm)]/Co (0.45 nm)/AlO_x (2 nm) sample. Supplementary Figure 3a shows the schematic illustration of the ST-FMR measurement [S7,S8], where a sample with dimensions of 10 μm × 10 μm was used. Note that all measurement conditions except for θ are identical to those for the measurement shown in Fig. 2 of the main text. We used a fixed azimuthal angle of magnetic field of 70°. Supplementary Figure 3b shows the normalized ST-FMR spectra for different polar angles between θ = 5° and θ = 90° with 5° steps. Here, we fixed the measurement frequency of 7 GHz. The extracted resonance field (B_{res}) from Supplementary Fig. 1b was plotted as a function of θ in Supplementary Fig. 3c. The solid line in Supplementary Fig. 3c is the fitting curve using the angular dependent Kittel equation [S9]. As θ increases, the B_{res} deviates from the Kittel equation, which is partly attributed to the TMS that occurs when a magnetic field is applied near an in-plane direction [S10]. This experiment indicates that the TMS is present in our sample, which can cause a resonance field shift under a near-in-plane magnetic field.

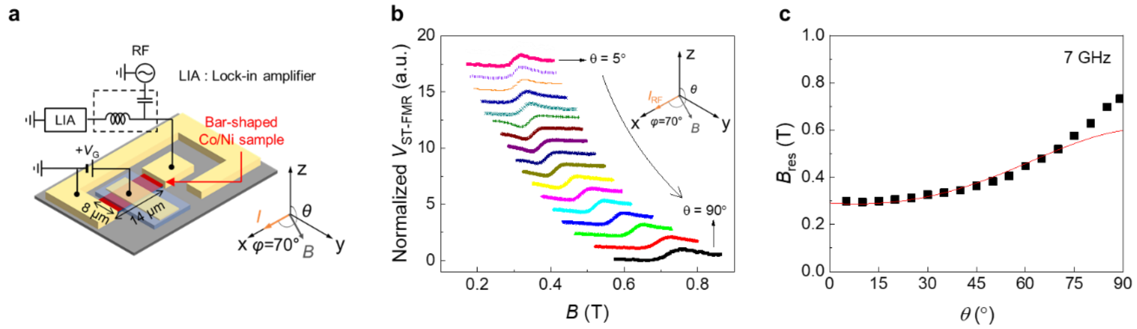

Supplementary Figure 3| ST-FMR measurement of Co/Ni sample for various direction of magnetic field. a, Schematic diagram of the device structure of the ST-FMR measurement set-up. **b,** Normalized ST-FMR spectra at 7 GHz depending on the polar angle (θ) of the magnetic field varying between 5° and 90° with 5° steps. **c,** Resonance field (B_{res}) as a function of θ extracted from Supplementary Fig. 3b. The solid line is a fit to the experimental data point using the Kittel equation associated with magnetic anisotropy.

Note 4. ST-FMR spectra for various frequencies

Supplementary Figure 4a shows the schematic illustration of the ST-FMR measurement [S7,S8], of which details are discussed in the Method section of the main text. Supplementary Figures 4b-4d show the ST-FMR spectra of the Co/Ni sample with different V_g's for various frequencies ranging from 10 to 21 GHz, which were measured while sweeping magnetic fields along the *z*-direction.

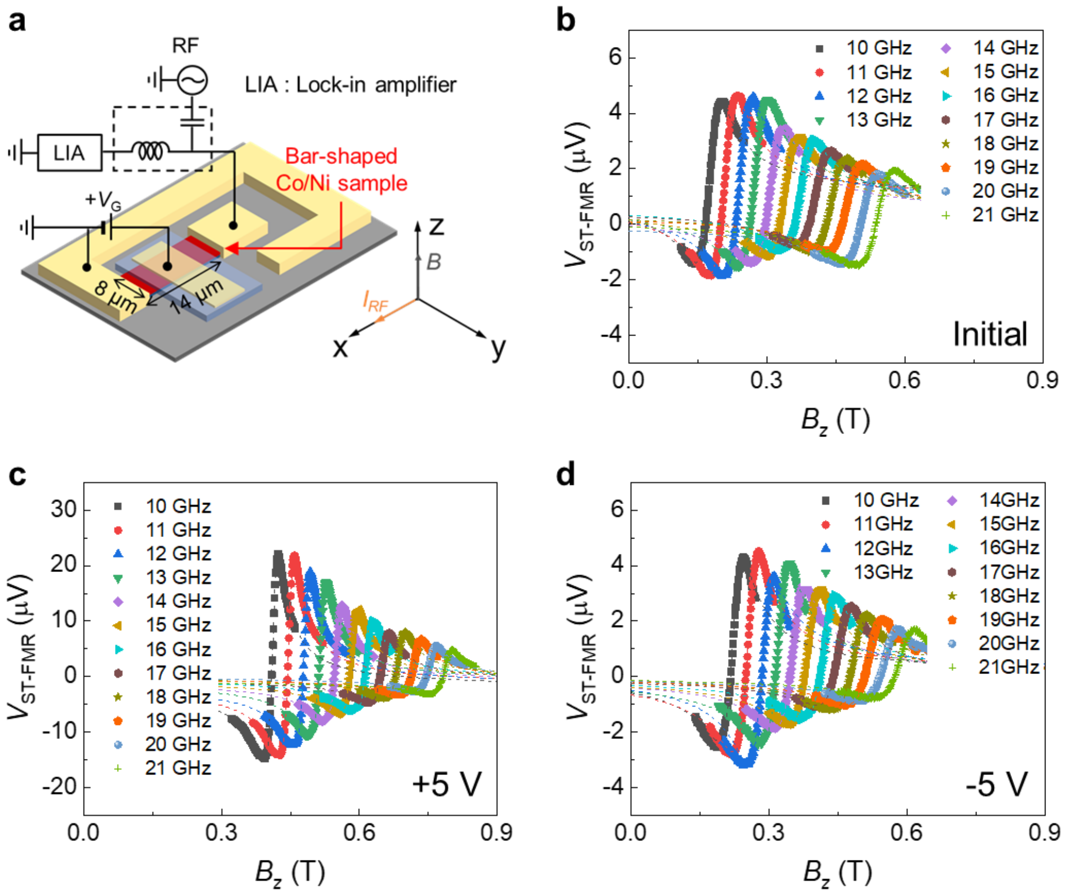

Supplementary Figure 4| ST-FMR measurement of Co/Ni sample for various frequencies. a, Schematic diagram of the device structure of the ST-FMR measurement set-up. **b-d,** ST-FMR spectra of the Co/Ni sample for various frequencies ranging from 10 to 21 GHz with sequentially applied gate voltages V_g= 0 V (initial) (**b**), V_g= +5 V (**c**), and V_g= −5 V (**d**). The dotted lines are the fitting curves based on Eq. (2) of the main text.

Note 5. Origin of voltage controlled magnetic anisotropy and damping

To verify the governing mechanism of the voltage-controlled magnetic anisotropy (VCMA) effect, we performed the following additional experiments: (i) measurement of resistance variation when a gate voltage is applied, (ii) comparison of the anisotropy changes by gate voltage application and oxidation time. As demonstrated below, these experimental results, along with the non-volatile nature, corroborate that the VCMA effect in our work is primarily due to the voltage-driven O^{2-} ion migration.

(i) Resistance variation by gate voltages

To verify the mechanism of VCMA, we measure the resistance change of the SHNO device during the cumulative voltage-driven frequency tuning experiment. It is expected that the voltage-driven O^{2-} ion migration induces resistance changes through oxidation or reduction at the interface. To confirm this, we repeated the same measurement shown in Fig. 5 of the main text with a dc current (I_{dc}) of 2.1 mA and a voltage (V_{g}) duration time of 30 s at room temperature. The top and bottom panels of Supplementary Fig. 5a show the auto-oscillation frequency change with the number of V_{g} pulses ($N_{V_{\mathrm{g}}}$) of +7 V and −5 V, respectively. The oscillation frequency gradually increases (decreases) by about 1.5 GHz with increasing $N_{V_{\mathrm{g}}}$ of +7 V (−5V), which is consistent with the result shown in Fig. 5a of the main text. While measuring the oscillation frequency with V_{g}, we monitored the resistance change $\frac{\Delta R}{R(0)}$ of the SHNO device. Here, $\frac{\Delta R}{R(0)}(\%) = \frac{R(N_{V_{\mathrm{g}}})-R(0)}{R(0)} \times 100,$ where $R(0)$ is the initial resistance and $R\left(N_{V_{\mathrm{g}}}\right)$ is the resistance after applying $N_{V_{\mathrm{g}}}$. Supplementary Figure 5b shows $\frac{\Delta R}{R(0)}$ versus $N_{V_{\mathrm{g}}}$ for V_{g}'s of +7 V and −5 V. When the negative (positive) V_{g} is applied, the ΔR increases (decreases), which suggests that the negative (positive) voltage drives the O^{2-} ion toward (away from) the ferromagnet/oxide interface, causing oxidation (reduction) of the ferromagnet at the interface. We remark that the sample resistance is fully restored to the initial value when the opposite voltage is applied, which indicates that the resistance variation is not caused by sample degradation, for example by Joule heating.

(ii) Comparison of the anisotropy changes by gate voltage application and by oxidation time

To further verify the VCMA mechanism, we measured the anisotropy variation with plasma oxidation time. Note that the negative voltage enhances the PMA of our Co/Ni film (Fig. 1c of the main text). To investigate whether the enhancement of the PMA is due to oxidation of ferromagnet at the interface, we prepared two Co/Ni samples with the same structure, but different O_2 plasma oxidation times (t = 25s, 125s). Supplementary Figure 6a shows the hysteresis curves of the two Co/Ni samples with different oxidation states the ferromagnet/oxide interface. The sample with longer oxidation has a larger coercivity field than the sample with shorter oxidation, suggesting that the PMA increases with oxidation time.

The variation of PMA with oxidation time is further evaluated by ST-FMR measurements. Supplementary Figure 6b shows the FMR frequency ($f_{\mathrm{res},z}$) as a function of resonance field $B_{\mathrm{res},z}$ for the samples, from which we extracted the anisotropy field, B_{k} of each sample. Supplementary Figure 6c demonstrates the results that the sample with longer oxidation shows a larger B_{k} than the sample with shorter oxidation. This shows that the longer (shorter) oxidation has the same effect on the PMA as the negative (positive) voltage, corroborating that the VCMA effect in our sample is primarily dominated by the voltage-driven O^{2-} ion migration.

As explained above, the gate voltage modifies the interfacial oxidation states by the voltage-induced O^{2-} ion migration. This would change the effective magnetic damping possibly through the modulation of interfacial Rashba spin-orbit-coupling (RSOC) as the surface (or interface) oxidation increases the RSOC [S11] and the RSOC modifies the damping [S12,S13]. We further check whether interfacial oxidation alters the effective magnetic damping in our sample. Supplementary Figure 7a shows the linewidth of the ST-FMR spectra for the samples with different oxidation times, which was obtained from the measurements shown in Supplementary Fig. 6b. As summarized in Supplementary Fig. 7b, the effective damping constant α_{eff} of the sample with longer oxidation is larger than that of the sample with shorter oxidation. This demonstrates that the enhancement in effective magnetic damping is due to the interface oxidation, possibly through the modulation of interfacial RSOC.

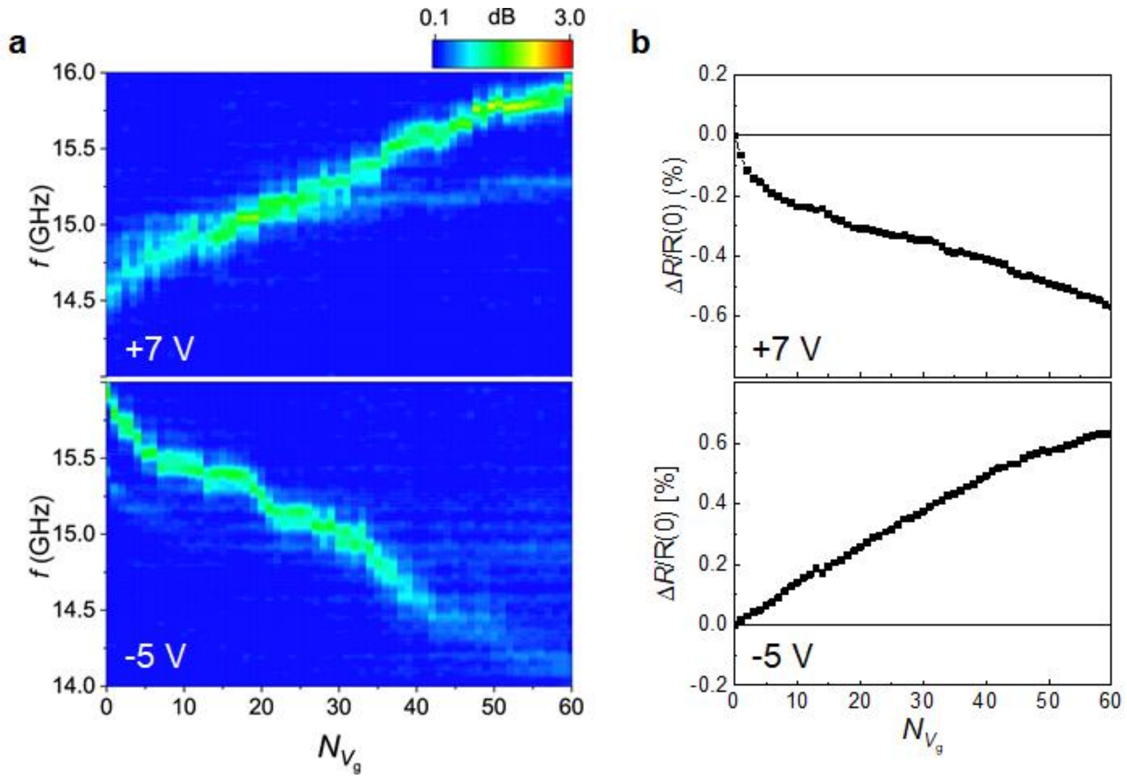

Supplementary Figure 5| Cumulative change in auto-oscillation frequency and device resistance. a, The color plots of power spectral density as a function of the number of V_{g} pulses ($N_{V_{\mathrm{g}}}$) of +7 V and −5 V, respectively. **b,** Resistance change ($\Delta R/R(0)$) versus the number of V_{g} pulses ($N_{V_{\mathrm{g}}}$) of +7 V and −5 V, respectively.

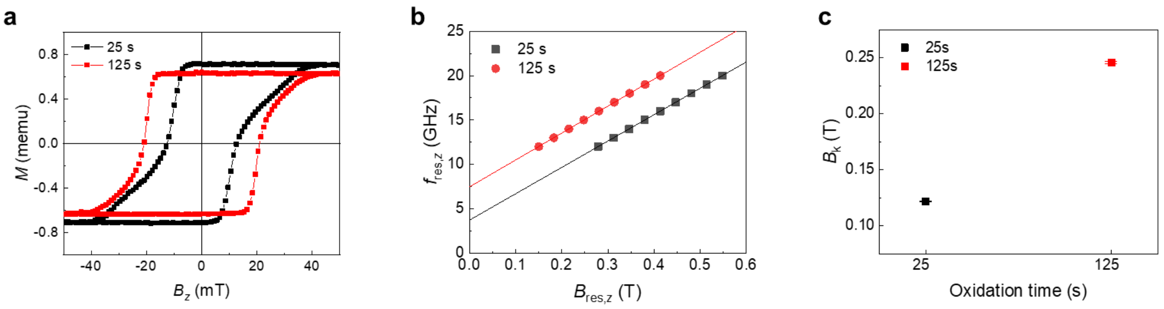

Supplementary Figure 6| Change of perpendicular magnetic anisotropy depending on plasma oxidation time. a, Magnetization hysteresis loops of two Co/Ni samples with out-of-plane magnetic field. Here, two samples have different plasma oxidation time, t = 25 s (black) and t = 125 s (red). **b,** FMR frequency as a function of resonance field ($B_{\mathrm{res},z}$) for the two samples. The solid lines are the best fit based on the Kittel formula, $f_{\mathrm{res},z} = \frac{\gamma}{2\pi}(B_{\mathrm{res},z} + B_{\mathrm{k}})$, where the γ is gyromagnetic ratio. **c,** Extracted anisotropy field as a function of plasma oxidation time. The error bars are due to the uncertainty in fitting the date in Supplementary Fig. 6b to the Kittel formula, $f_{\mathrm{res},z} = \frac{\gamma}{2\pi}(B_{\mathrm{res},z} + B_{\mathrm{k}})$.

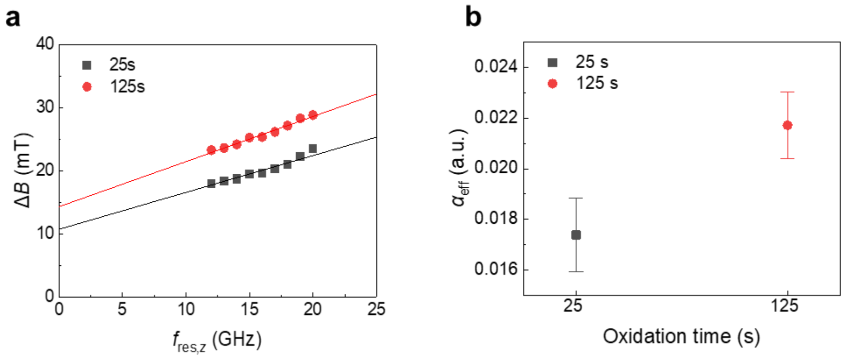

Supplementary Figure 7| Change of effective damping constant depending on plasma oxidation time. a, Line width of ST-FMR spectrum as a function of driving frequency for two Co/Ni samples having different plasma oxidation time, t = 25 s (black) and t = 125 s (red). Solid lines are the best fit based on Kittel formula, $\frac{d\Delta B}{df} = \frac{2\pi\alpha_{\mathrm{eff}}}{\gamma}$, where the γ is gyromagnetic ratio. **b,** Effective magnetic damping constant as a function of plasma oxidation time, extracted from Supplement Fig. 7a. The error bars are due to the uncertainty in fitting the data in Supplementary Fig. 7a to the equation, $\frac{d\Delta B}{df} = \frac{2\pi\alpha_{\mathrm{eff}}}{\gamma}$.

Note 6. Voltage-driven frequency modulation in Co/Ni sample with a different thickness

To show the reproducibility of the voltage-driven frequency modulation, we fabricated another Co/Ni device of Ta (3 nm)/Pt (5 nm)/[Co (0.4 nm)/Ni (0.6 nm)]$_7$/Co (0.4 nm)/AlO_x (2 nm), where a slightly thinner Co (0.4 nm) is used compared to the sample used in the main text has a [Ta (3 nm)/Pt (5 nm)/[Co (0.45 nm)/Ni (0.6 nm)]$_7$/Co (0.45 nm)/AlO_x (2 nm)]. We first check PMA of the sample using the ST-FMR measurement with the same procedure used in Fig. 2 of the main text. Supplementary Figure 8 shows the resonance frequency ($f_{\mathrm{res,z}}$) of ST-FMR spectra as a function of the resonance field ($B_{\mathrm{res,z}}$) for two Co/Ni samples having different Co thicknesses. As the y-intercept indicates the PMA field (B_{k}) according to the Kittel formula $f_{\mathrm{res,z}} = \frac{\gamma}{2\pi}(B_{\mathrm{res,z}} + B_{\mathrm{k}})$ [S14], demonstrating that the sample with a thinner Co has a stronger PMA.

We then fabricate a SHNO with a constriction width of 100 nm. The experimental procedure for the power spectral density (PSD) measurement is the same as used in Fig. 3 of the main text, except for a dc current (I_{dc}) of 1.8 mA used. Supplementary Figures 9a-9c show the color plots of PSD as a function of a magnetic field (B), where gate voltages of +5 V and −3V were sequentially applied. The auto-oscillation peak is clearly observed, and its frequency is increased by the positive voltage and restored by the subsequent negative voltage. Supplementary Figure 9d shows the auto-oscillation spectra for a magnetic field of B= 0.9 T for different gate voltages, extracted from Supplementary Figs. 9a-9c. The frequency modulation of the sample with a thinner Co is about a few GHz, confirming the reproducibility of the voltage-driven frequency modulation of the SHNO.

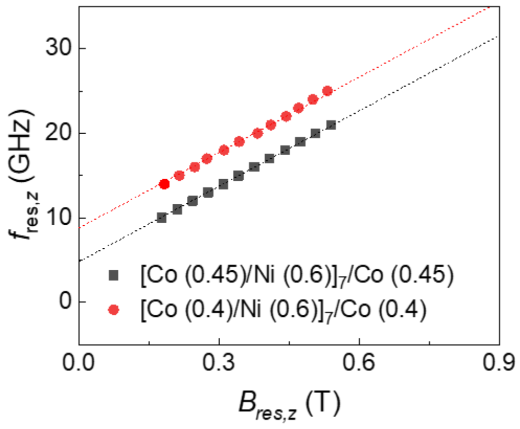

Supplementary Figure 8| ST-FMR measurement. Resonance frequency ($f_{res,z}$) of ST-FMR spectra as a function of the resonance field ($B_{res,z}$) for the samples of Ta (3 nm)/Pt (5 nm)/[Co (0.45 nm)/Ni (0.6 nm)]$_7$/Co (0.45 nm)/AlO_x (2 nm) (black squares) and Ta (3 nm)/Pt (5 nm)/[Co (0.4 nm)/Ni (0.6 nm)]$_7$/Co (0.4 nm)/AlO_x (2 nm) structures (red circles).

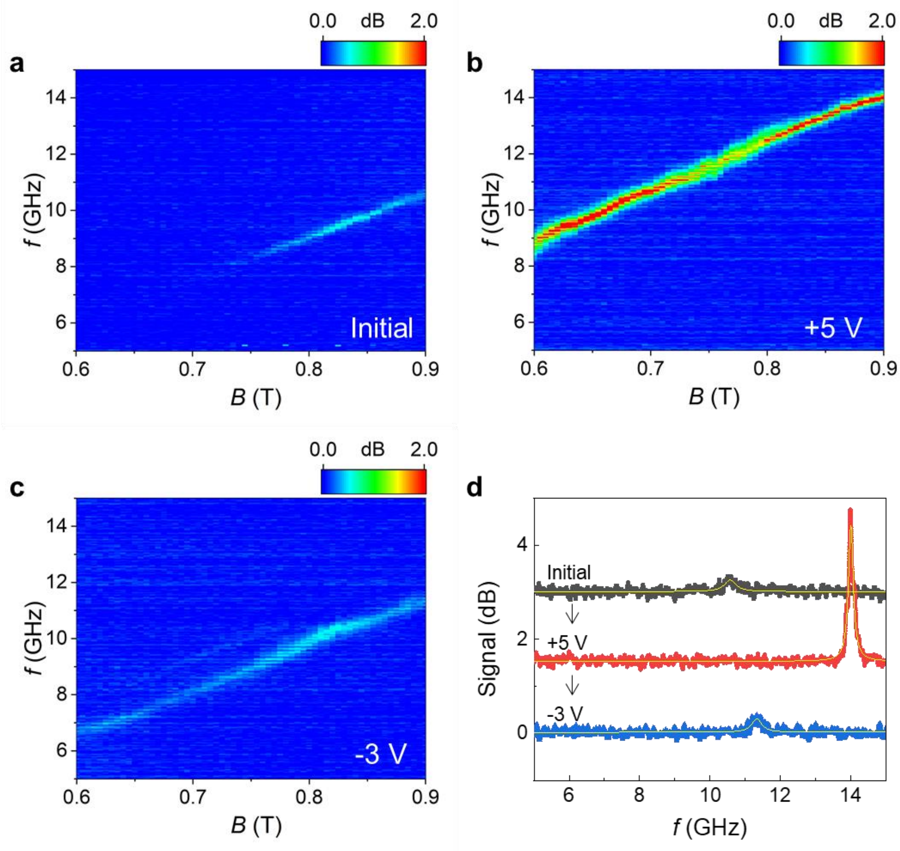

Supplementary Figure 9| Voltage-driven frequency modulation in SHNO of Ta (3 nm)/Pt (5 nm)/[Co (0.4 nm)/Ni (0.6 nm)]$_7$/Co (0.4 nm)/AlO$_x$ (2 nm). a-c, PSDs versus a magnetic field for sequentially applied gate voltages, V_g= 0 V (initial state) (**a**), V_g= +5 V (**b**), and V_g= −3 V (**c**). I_{dc}= 1.8 mA. **d,** Auto-oscillation spectra for B= 0.9 T for different gate voltages, extracted from Supplementary Figs. 9a-9c. The yellow line are the Lorentz fits of the auto-oscillation spectra.

Note 7. Gate voltage effect on the current-induced SOT

We investigated the gate voltage effect on current-induced spin-orbit torque (SOT) using in-plane harmonic measurements. For the measurement, we fabricated a Hall bar device with a 10 μm × 10 μm cross using the Ta (3 nm)/Pt (5 nm)/[Co (0.45 nm)/Ni (0.6 nm)]$_7$/Co (0.45 nm)/AlO_x (2 nm) film. The first and second harmonic Hall resistance ($R_{1\omega}$ and $R_{2\omega}$) were simultaneously measured with an ac current of 15 mA and a frequency of 11 Hz while rotating the sample (azimuthal angle φ) under an in-plane magnetic field ($B_{\rm ext}$). The $B_{\rm ext}$ ranges from 0.3 T to 4.0 T, which is larger than the perpendicular magnetic anisotropy field ($B_{\rm k}$), so the magnetization is aligned in the magnetic field direction. The gate voltage was applied to the top electrode for 5 minutes at 150 °C before the measurement. Supplementary Figure 10a shows the $R_{2\omega}$ as a function of φ under a magnetic field of 0.6 T for various gate voltages. The $R_{2\omega}$ can be expressed as [S15,S16],

$$R_{2\omega}(\varphi) = \left(R_{\rm AHE}\frac{B_{\rm DLT}}{B_{\rm eff}} + R_{\nabla T}\right)\cos\varphi + 2R_{\rm PHE}\frac{B_{\rm FLT}}{B_{\rm ext}}(2\cos^3\varphi - \cos\varphi), \qquad \text{(S1)}$$

where $R_{\rm AHE}$, $R_{\rm PHE}$ and $R_{\nabla T}^{2\omega}$ are the anomalous Hall resistance, planar Hall resistance and thermal effect contribution, respectively; $B_{\rm DLT}$ and $B_{\rm FLT}$ are the damping-like effective field and field-like effective field including Oersted field, respectively; $B_{\rm eff}$ is the effective magnetic field ($B_{\rm eff} = B_{\rm ext} - B_{\rm k}$). Supplementary Figures 10b-10c show the magnetic field-dependence of the $\cos\varphi$ and ($2\cos^3\varphi - \cos\varphi$) components of $R_{2\omega}$, respectively. Supplementary Figure 10d demonstrates that the extracted $B_{\rm DLT}$ and $B_{\rm FLT}$ values of the sample, which are not changed by the gate voltage.

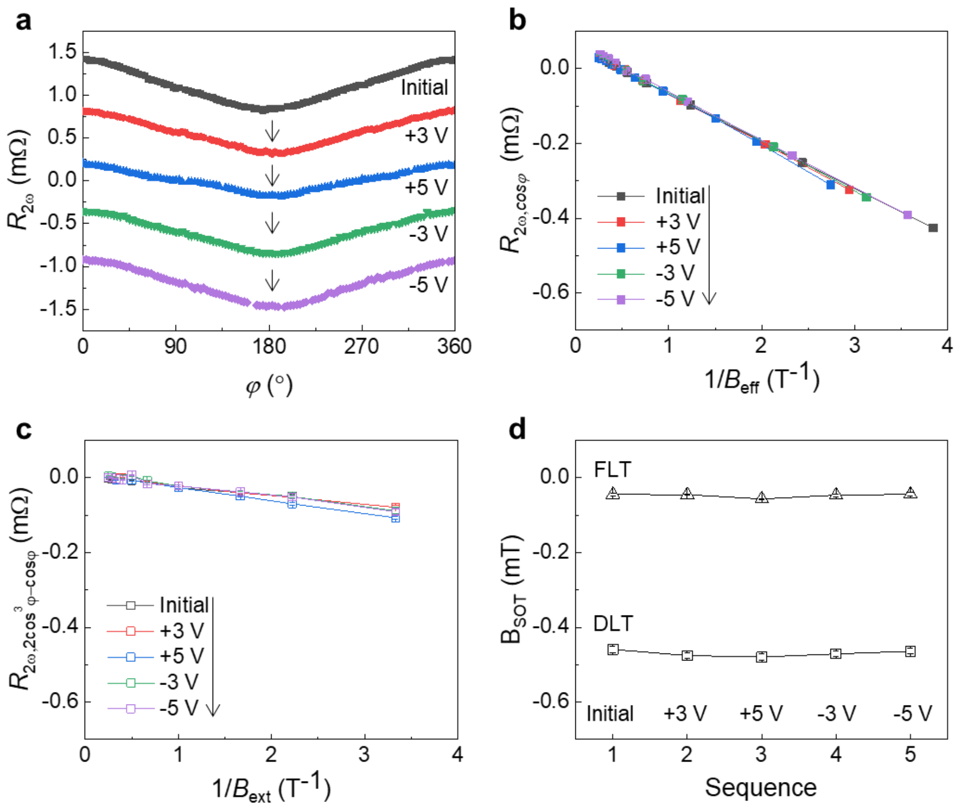

Supplementary Figure 10| Gate voltage effect on current-induced SOT. a, Second harmonic Hall resistance $R_{2\omega}$ versus azimuthal angle φ under an in-plane magnetic field of 0.6 T for sequentially applied gate voltages. **b,** $\cos\varphi$ component of $R_{2\omega}$ as a function of $1/B_{\mathrm{eff}}$ for sequentially applied gate voltages. **c,** $(2\cos^3\varphi - \cos\varphi)$ component of $R_{2\omega}$ as a function of $1/B_{\mathrm{ext}}$ for sequentially applied gate voltages. The gate voltages were applied in the sequence indicated by the black arrows in Supplementary Figs. 10a-10c. **d,** The variation of the SOT-induced B_{DLT} (black square) and B_{FLT} (black triangle) values with sequentially applied gate voltages. All error bars are due to the uncertainty in fitting the data in Supplementary Figs. 10b-10c to the Eq. (S1).

Note 8. Nonlinearity coefficient depending on perpendicular magnetic anisotropy

The nonlinearity coefficient (N) of the auto-oscillation depends on the perpendicular magnetic anisotropy field (B_k) [S17,S18]. However, we did not observe a significant change in the current-dependent frequency, although the B_k changes by 0.24 T with the gate voltage. We attribute the weak nonlinearity to the large polar angle $\theta = 80^o$ used for our measurements. To verify this argument, we calculate the N value based on the equation used in the literature with our experimental parameters [S19]. Supplementary Figure 11 shows the mapping of the N values as functions of B_k and external magnetic field (B_{ext}) with different polar angles (θ). The black lines represent the range of B_k change due to the VCMA effect extracted from Fig. 2 of the main text. When θ = 10° (Supplementary Fig. 11a), the N varies largely with B_k, consistent with results in the literature [S18]. On the other hand, for θ = 80° (Supplementary Fig. 11b, our experimental condition), the change in N caused by the VCMA effect is considerably reduced compared to that for θ = 10°. Note that this result shows the trend of the non-linearity versus the measurement angle, but it cannot quantitatively explain the difference in the non-linearity between the two experiments.

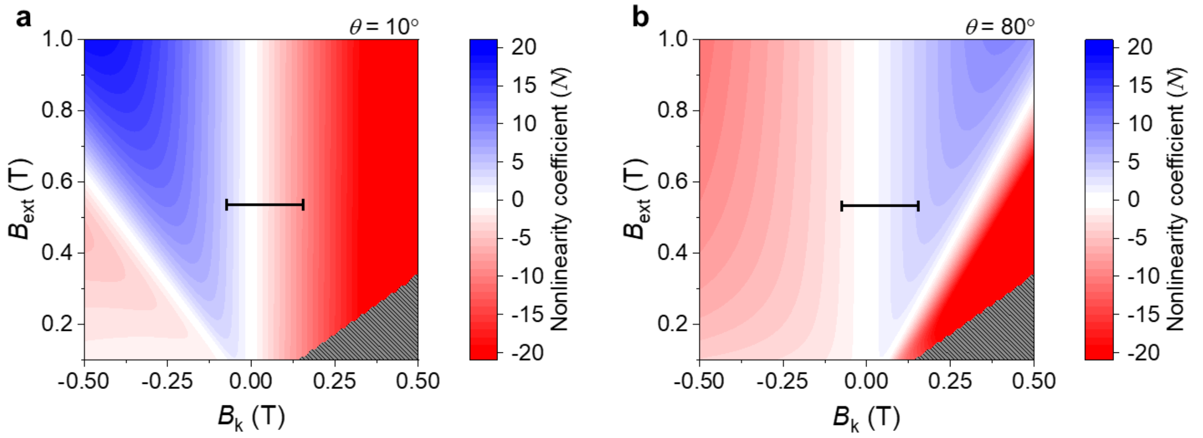

Supplementary Figure 11| Nonlinearity coefficient depending on the field geometry. a-b, Calculated nonlinearity coefficient (N) as functions of perpendicular magnetic anisotropy field (B_k) and external magnetic field (B_{ext}) with a different polar angle (θ) of magnetic field, θ = 10° (**a**), θ = 80° (**b**). The black bar in each figure represents the area of B_k change by VCMA (B_k = –0.08 T ~ 0.16 T) at the magnetic field where the auto-oscillation spectra were measured.

Note 9. Comparison of auto-oscillation frequency and FMR frequency

There are three different modes in SHNO; propagating spin wave mode, localized ‘bullet’ mode, and quasi-propagating mode. According to the literatures [S20], the propagating spin wave mode appears at a low current regime, and as the current increases, the localized ‘bullet’ mode starts to appear in an intermediate current regime and finally, the quasi-propagating mode that is weakly localized due to Oersted field is dominant at a high current regime. These three modes can be distinguished by comparing the auto-oscillation frequency (f_{AO}) and ferromagnetic resonance frequency (f_{res}); the propagation spin wave mode (localized mode) is dominant for $f_{\mathrm{AO}} > f_{\mathrm{res}}$ ($f_{\mathrm{AO}} < f_{\mathrm{res}}$) [S19,S20]. Here we provide the detailed comparison between f_{AO} and f_{res}.

To verify the spin wave mode in our sample, we compare the FMR and auto oscillation frequencies measured using the same sample with identical conditions. In this regard, we first measured auto-oscillation power spectral density using a 100 nm-constriction SHNO device of a Ta (3 nm)/Pt (5 nm)/[Co (0.45 nm)/Ni (0.6 nm)]$_7$/Co (0.45 nm)/AlO_x (2 nm) structure. Supplementary 12a shows the auto-oscillation power spectral density as a function of the magnetic field at a dc current of 2.3 mA. Here, the polar (θ) and the azimuthal angles (φ) of the applied magnetic field are 80° and 70°, respectively. We also performed the ST-FMR measurement using the same device under the same magnetic field direction. Note that the ST-FMR measurement was done with a microwave power of -17 dBm, but no dc current is applied. Supplementary Figure 12b shows the f_{res} of the ST-FMR as a function of the resonance field (B_{res}). Notably, the auto-oscillation frequency is higher than the ST-FMR f_{res} for the same magnetic field, which is opposite to those previously obtained from the calculations.

We further confirm this by measuring the ST-FMR while increasing a dc current. Supplementary Figure 12c shows the results. For small currents (0 ~ 1.9 mA), only one resonance peak appears near ~0.70 T, which corresponds to the FMR resonance field. For large current (~2.3 mA), on the other hand, another peak appears near ~0.35 T, which is attributed to auto-oscillation. These results corroborate that the auto-oscillation frequency is higher than the FMR frequency. Therefore, the auto-oscillation in our device occurs in a propagation mode. This is consistent with positive nonlinearity of our SHNO device.

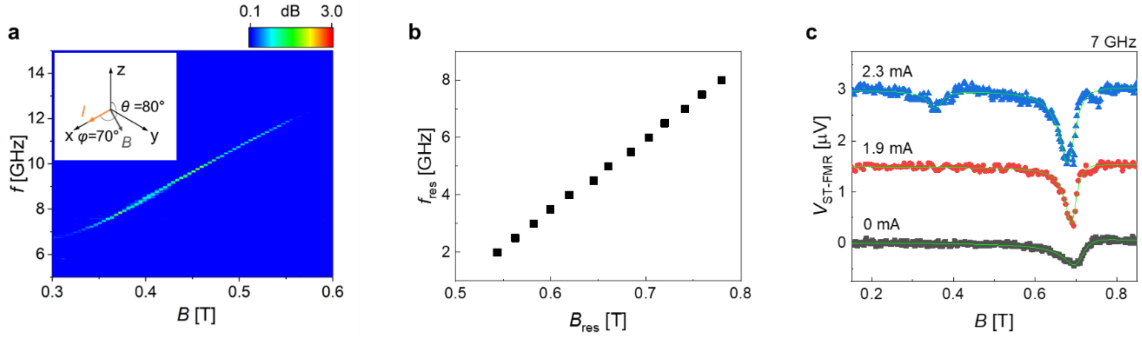

Supplementary Figure 12| Comparison of auto-oscillation frequency and ST-FMR frequency. a, The auto-oscillation power spectral density as a function of magnetic field at a dc current of 2.3 mA for a 100 nm-constriction SHNO device of a Ta (3 nm)/Pt (5 nm)/[Co (0.45 nm)/Ni (0.6 nm)]$_7$/Co (0.45 nm)/AlO_x (2 nm) structure. **b,** Resonance frequency (f_{res}) as a function of resonance field (B_{res}) without dc current. **c,** ST-FMR spectra for three different dc currents of 0, 1.9 mA, and 2.3 mA. θ= 80° and φ= 70°.

Note 10. Threshold current for current-induced magnetization auto-oscillation

We determine the threshold current (I_{th}) at which auto-oscillation begins to occur by a linear fit of the inverse of the PSD integral. Supplementary Figures 13a-13e show the (integral of PSD)$^{-1}$ versus current for sequentially applied gate voltages, where I_{th} is obtained by the *x*-intercept of the linear fit (red solid lines) [S21,S22]. Supplementary Figure 13f displays the variation of I_{th} with gate voltages, which is the same as Fig. 4f in the main text.

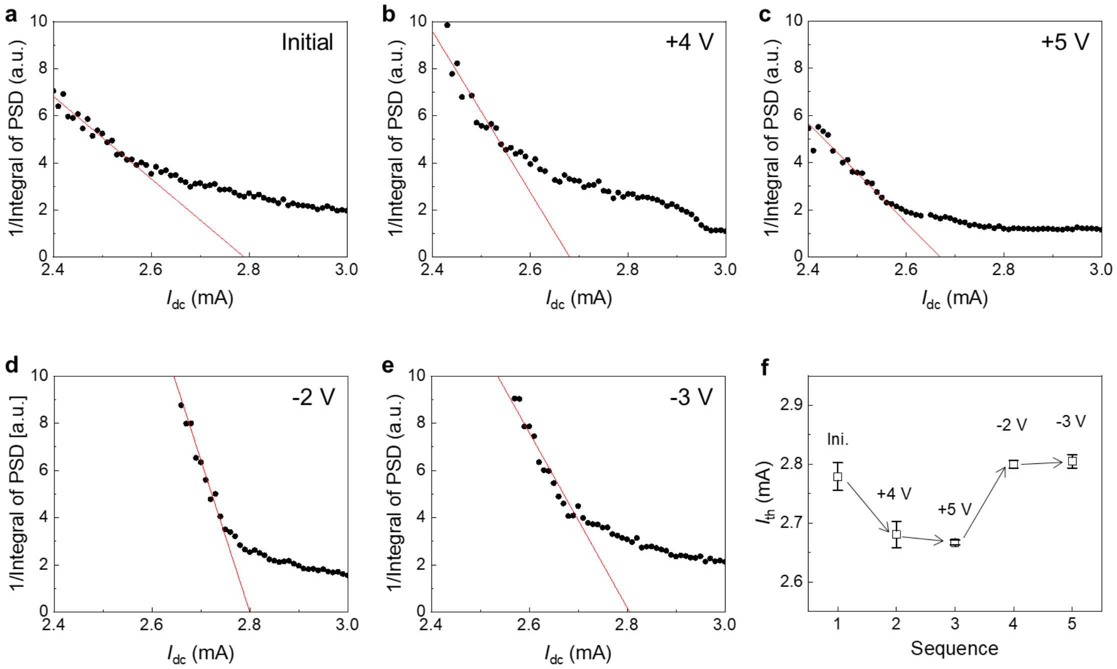

Supplementary Figure 13| Threshold current for current-induced magnetization auto-oscillation. a-e, (integral of PSD)$^{-1}$ as a function of current for sequentially applied gate voltages, V_g= 0V (initial) (**a**), V_g= +4 V (**b**), V_g= +5 V (**c**), V_g= −2 V (**d**), and V_g= −3 V (**e**). B = 0.56 T. **f,** I_{th} according to the sequentially applied gate voltages, extracted from Supplementary Figs. 13a-13e. The error bars are due to the uncertainty in the linear fit of the data in Supplementary Figs. 13a-13e.

Note 11. Gate-induced leak current

We measured the leak current (I_{leak}) as a function of gate voltage (V_g) in the Ta (3 nm)/Pt (5 nm)/[Co (0.45 nm)/Ni (0.6 nm)]$_7$/Co (0.45 nm)/AlO_x (2 nm)/Ta(3 nm)/ZrO_2(40 nm) structure, which is the same sample shown in Fig. 1 of the main text. Supplementary Figure 14 shows the *I-V* curve, demonstrating that I_{leak} is less than 3 pA within our experimental V_g range of −5 V to +5 V. Since the I_{leak} is 10^9 times smaller than the threshold current for auto-oscillation of a few mA, we believe that the leak current does not significantly affect the device properties nor induce Joule heating

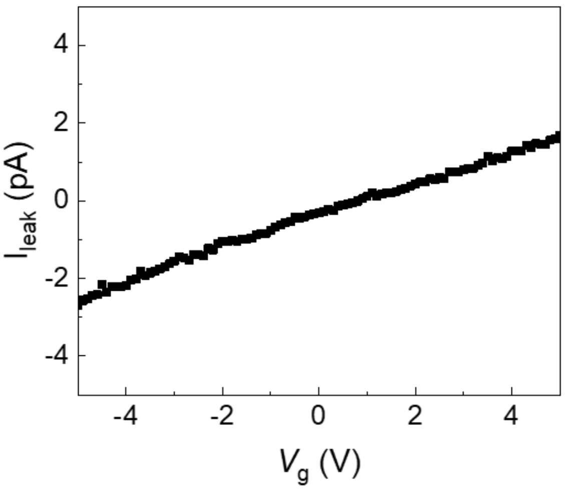

Supplementary Figure 14| The leakage current I_{leak} due to the gate voltage V_g for the Ta (3 nm)/Pt (5 nm)/[Co (0.45 nm)/Ni (0.6 nm)]$_7$/Co (0.45 nm)/AlO_x (2 nm)/Ta(3 nm)/ZrO_2(40 nm) structure.

References

[S1] Yen, T. J., Gismatulin, A., Volodin, V., Gritsenko V. & Chin, A. All Nonmetal Resistive Random Access Memory. *Sci. Rep.* **9**. 6144 (2019).

[S2] Yen, T. J., Chin, A. & Gritsenko, V. High Performance All Nonmetal SiN_x Resistive Random Access Memory with Strong Process Dependence. *Sci. Rep.* **10**, 2807 (2020).

[S3] Jeon, K. et al. Self-rectifying resistive memory in passive crossbar arrays. *Nat. Commun.* **12**, 2968 (2021)

[S4] Bliss, T. V. & Collingridge, G. L. A synaptic model of memory: long-term potentiation in the hippocampus. *Nature* **361**, 31-39 (1993).

[S5] Abbott, L. F. & Regehr, W. G. Synaptic computation. *Nature* **431**, 796-803 (2004).

[S6] Kang, M. -G. et al. Electric-field control of field-free spin-orbit torque switching via laterally modulated Rashba effect in $Pt/Co/AlO_x$ structures. *Nat. Commun.* **12**, 7111 (2021)

[S7] Liu, L., Moriyama, T., Ralph, D. C. & Buhrman, R. A. Spin-torque ferromagnetic resonance induced by the spin Hall effect. *Phys. Rev. Lett.* **106**, 306601 (2011).

[S8] Kim, J. H. et al. Spin-orbit torques associated with ferrimagnetic order in Pt/GdFeCo/MgO layers. *Sci. Rep.* **8**, 6017 (2018).

[S9] Sukhov, A. et al. Angular dependence of ferromagnetic resonance as indicator of the nature of magnetioelectric coupling in ferromagnetic-ferroelectric heterostructures. *Phys. Rev. B* **90**, 224428 (2014).

[S10] Beaujour, J. -M. el al. Ferromagnetic resonance linewidth in ultrathin films with perpendicular magnetic anisotropy. *Phys. Rev. B* **80**, 180415(R) (2009).

[S11] Krupin, O. et al. Rashba effect at magnetic metal surfaces. *Phys. Rev. B* **71**, 201403(R) (2005).

[S12] Kim, K.-W., Moon, J.-H., Lee, K.-J. & Lee, H.-W. Prediction of Giant Spin Motive Force due to Rashba Spin-Orbit Coupling. *Phys. Rev. Lett.* **108**, 217202 (2012).

[S13] Tatara, G., Nakabayashi, N. & Lee, K.-J. Spin motive force induced by Rashba

interaction in the strong sd coupling regime. *Phys. Rev. B* **87**, 054403 (2013).

[S14] Beaujour, J.-M., Ravelosona, D., Tudosa, I., Fullerton, E. E. & Kent, A. D. Ferromagnetic resonance linewidth in ultrathin films with perpendicular magnetic anisotropy. *Phys. Rev. B* **80**, 180415(R) (2009).

[S15] Hayashi, M., Kim, J., Yamanouchi, M. & Ohno, H. Quantitative characterization of the spin-orbit torque using harmonic Hall voltage measurements. *Phys. Rev. B* **89**, 144425 (2014).

[S16] Avci, C. O. et al. Interplay of spin-orbit torque and thermoelectric effects in ferromagnet/normal-metal bilayers. *Phys. Rev. B* **90**, 224427 (2014).

[S17] Slavin, A. & Tiberkevich, V. Nonlinear auto-oscillator theory of microwave generation by spin-polarized current. *IEEE Trans. Magn.* **45**, 1875–1918 (2009).

[S18] Fulara, H., et al. Spin-orbit torque-driven propagating spin waves. *Sci. Adv.* **5**, eaax8467 (2019).

[S19] Mohseni, M. et al. Magnetic droplet soliton nucleation in oblique fields. *Phys. Rev. B* **97**, 184402 (2018).

[S20] Chen, L., Urazhdin, S., Du, Y. W. & Liu, R. H. Dynamical Mode Coupling and Coherence in a Spin Hall Nano-Oscillator with Perpendicular Magnetic Anisotropy. *Phys. Rev. Appl* **11**, 064038 (2019).

[S21] Tiberkevich, V., Slavin, A. & Kim, J. Von. Microwave power generated by a spin-torque oscillator in the presence of noise. *Appl. Phys. Lett*. **91**, 192506 (2007).

[S22] Awad, A. A., Houshang, A., Zahedinejad, M., Khymyn, R. & Åkerman, J. Width dependent auto-oscillating properties of constriction based spin Hall nano-oscillators. *Appl. Phys. Lett*. **116**, 232401 (2020).
